# 1399. Pancreatic Abscess in Disseminated Melioidosis

**DOI:** 10.1093/ofid/ofad500.1236

**Published:** 2023-11-27

**Authors:** Yen Tsen Saw, Heng Gee Lee, Kylie Sze Tyng Yong, Suet Hwa Kok

**Affiliations:** Queen Elizabeth Hospital, Kota Kinabalu, Sabah, Malaysia; Queen Elizabeth Hospital, Kota Kinabalu, Sabah, Malaysia; Queen Elizabeth Hospital, Kota Kinabalu, Sabah, Malaysia; Queen Elizabeth Hospital, Kota Kinabalu, Sabah, Malaysia

## Abstract

**Background:**

Melioidosis is an infection caused by the gram-negative bacterium *Burkholderia pseudomallei* that is widely distributed in the tropical regions of Southeast Asia. It is commonly associated with intraabdominal abscesses, particularly the liver and spleen. However, there is scarce literature regarding pancreatic involvement in melioidosis. We describe a case series of pancreatic manifestations of disseminated melioidosis in a tertiary hospital in Sabah, Malaysia.

**Methods:**

We conducted a retrospective case review of 63 patients with culture-positive melioidosis admitted in Queen Elizabeth Hospital, Sabah between December 2021 and March 2023 and identified cases with pancreatic involvement diagnosed by radiological imaging.

**Results:**

Pancreatic involvement was reported in 3 patients (4.8%). Median duration of symptoms was 20 days. All three patients had risk factors for melioidosis (3 had poorly controlled diabetes mellitus and 1 had chronic kidney disease), and had infection involving other organs (lungs, liver, spleen and prostate). Pancreatic lesions were all detected by computed tomography scans in the tail region and ranged from small microabscesses to large solitary lesions (largest dimension 5.6 cm) with peripancreatic fat stranding. One patient required emergency hemodialysis and intensive care. Drainage of concurrent prostatic abscess was done for one patient. All 3 patients were treated with antibiotics alone (median 38 days of intensive therapy) without drainage of pancreatic abscess and showed regression of pancreatic lesions upon reassessment imaging.

**Figure:**
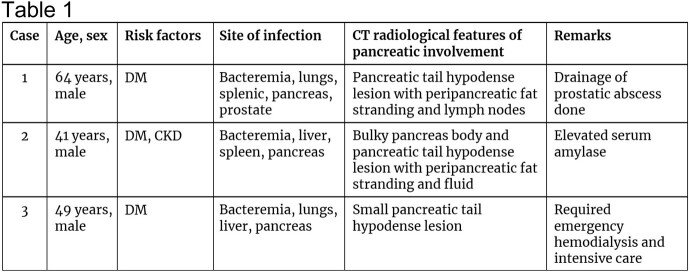

Patient demographic and disease details. DM, diabetes mellitus; CKD, chronic kidney disease; CT, computed tomography.

**Figure 1**

**Figure:**
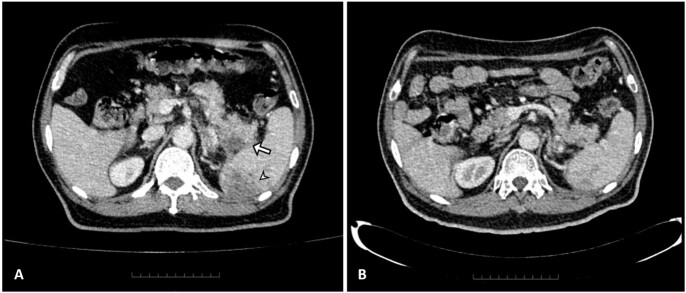

(A) Contrast-enhanced computed tomography image showing a heterogenous hypodense lesion at the pancreatic tail (arrow) and multiple ill-defined splenic lesions (arrowhead). (B) Smaller lesions at the pancreatic tail and spleen after 35 days of antibiotics (Case 1).

**Conclusion:**

Surgical drainage is required for source control of large melioidotic abscesses, however it may not be feasible for smaller pancreatic abscesses which are often multifocal and deep seated. In our study, the patients with pancreatic abscesses showed good clinical improvement with antibiotics therapy without drainage. Further studies are needed on the role of debridement for pancreatic melioidosis. Published guidelines recommend treatment of melioidosis with deep-seated abscesses with four weeks of intravenous ceftazidime as intensive therapy, followed by three to six months of oral trimethoprim-sulfamethoxazole or doxycycline as eradication therapy.

**Disclosures:**

**All Authors**: No reported disclosures

